# Depletion of Gangliosides Enhances Articular Cartilage Repair in Mice

**DOI:** 10.1038/srep43729

**Published:** 2017-03-02

**Authors:** Masatake Matsuoka, Tomohiro Onodera, Kentaro Homan, Fumio Sasazawa, Jun-ichi Furukawa, Daisuke Momma, Rikiya Baba, Kazutoshi Hontani, Zenta Joutoku, Shinji Matsubara, Tadashi Yamashita, Norimasa Iwasaki

**Affiliations:** 1 Department of Orthopaedic Surgery, Hokkaido University Graduate School of Medicine, Sapporo, Japan; 2 Laboratory of Biochemistry, Azabu University, Graduate School of Veterinary Medicine, Sagamihara, Japan

## Abstract

Elucidation of the healing mechanisms in damaged tissues is a critical step for establishing breakthroughs in tissue engineering. Articular cartilage is clinically one of the most successful tissues to be repaired with regenerative medicine because of its homogeneous extracellular matrix and few cell types. However, we only poorly understand cartilage repair mechanisms, and hence, regenerated cartilage remains inferior to the native tissues. Here, we show that glycosylation is an important process for hypertrophic differentiation during articular cartilage repair. GM3, which is a precursor molecule for most gangliosides, was transiently expressed in surrounding damaged tissue, and depletion of GM3 synthase enhanced cartilage repair. Gangliosides also regulated chondrocyte hypertrophy via the Indian hedgehog pathway. These results identify a novel mechanism of cartilage healing through chondrocyte hypertrophy that is regulated by glycosylation. Manipulation of gangliosides and their synthases may have beneficial effects on articular cartilage repair.

Over 200 million people worldwide suffer from osteoarthritis (OA), which is characterized by the progressive breakdown of articular cartilage^1^,^2^ and ultimately leads to the functional failure of synovial joints. Because articular cartilage has a limited potential for regeneration, many attempts have been made to enhance cartilage healing to prevent OA pathogenesis^3^,^4^,^5^. However, the regenerated cartilage in these attempts is considered to be inferior compared with native cartilage tissue.

One reason for the difficulty in improving cartilage regeneration is our poor understanding of the mechanism of articular cartilage repair. Regarding OA pathogenesis, several mechanisms of articular cartilage degradation have been shown in OA mouse models^6^,^7^,^8^,^9^. In contrast, the molecular mechanism of articular cartilage repair is not fully understood because until recently, no appropriate mouse model was available. A cartilage healing mouse model has been established that allows analysis of the cartilage regeneration process^10^. Elucidation of the cartilage repair process is essential for optimal manipulation of articular cartilage regeneration.

Glycans regulate various metabolic pathways including those that are active in cartilage tissue^11^,^12^. Glycosphingolipids (GSLs) are a group of glycolipids that are widely distributed on vertebrate plasma membranes. These molecules form clusters on cell membranes where they modulate transmembrane signaling and mediate cell-to-cell and cell-to-matrix interactions^13^,^14^. GSLs comprise diverse types of glycolipids and are classified into several groups. Among these groups, gangliosides are the most abundant GSLs in cartilage^15^ and play roles in maintaining chondrocyte homeostasis^16^ and differentiation^17^. In addition, the total ganglioside content in OA cartilage is decreased by 40%^18^,^19^, suggesting that gangliosides play a dominant role in cartilage metabolism and differentiation.

Therefore, we hypothesize that gangliosides regulate cartilage differentiation and/or metabolism during the reparative process of damaged articular cartilage. To test this hypothesis, we first investigated the normal expression pattern of GM3, which is a precursor molecule for most complex ganglioside species. We subsequently employed a strain of mice that are genetically engineered to lack GM3 synthase (GM3S; GM3^−/−^ mice) and are practically ganglioside deficient. The final goal of the current study was to clarify the functional role of gangliosides in articular cartilage repair.

## Results

### The normal expression pattern of GM3 during the articular cartilage repair process and chondrogenic differentiation

**Figure 1:**
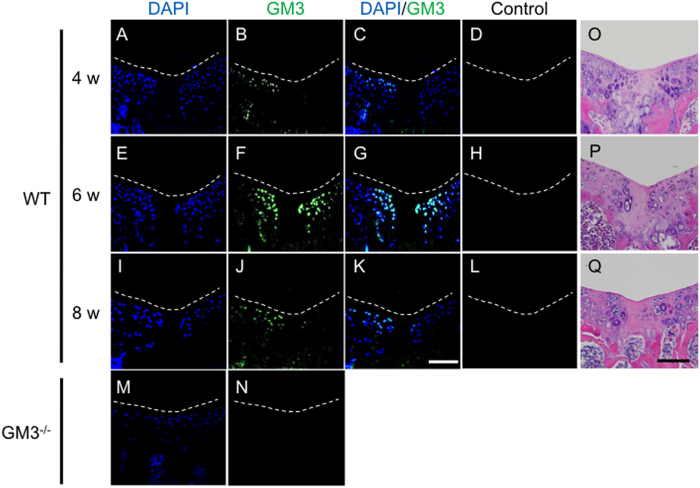
GM3 expression after osteochondral injury in WT mice (**A**–**L**). Four weeks (**A**–**D**), 6 weeks (**E**–**H**), and 8 weeks (**I**–**L**) after osteochondral injury. GM3 expression in 8-week-old GM3^−/−^ mice (**M**,**N**). White dotted lines show the margin of the patella groove. Representative histology with hematoxylin & eosin at 4 weeks (**O**), 6 weeks (**P**), and 8 weeks (**Q**). The scale bars show 100 μm.

**Figure 2:**
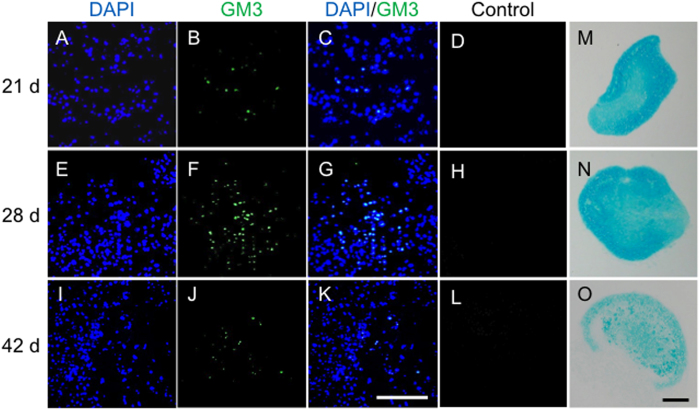
GM3 expression during chondrogenic differentiation (**A**–**L**). Day 21 (**A**–**D**), day 28 (**E**–**H**), day 42 (**I**–**L**). Representative histology with Alcian blue staining at 21 days (**M**), 28 days (**N**), and 42 days (**O**). The scale bars show 100 μm.

We previously reported that among the GSLs, the ganglio-series was most abundant in cartilage^15^. Here we analyzed the ganglio-series and showed that GM3, which serves as a precursor molecule for gangliosides, was the most abundant ganglioside (relative expression; 23%, Supplemental Fig. 1) in cartilage. Then we analyzed the localization of GM3 during the articular cartilage repair process after osteochondral injury (injury model) using wild-type (WT) mice^10^. GM3 was transiently increased surrounding the repaired tissue, with a peak 6 weeks postoperative (Fig. 1A–L), suggesting that GM3 plays important roles in the late phase of the articular cartilage repair process. In an *in vitro* experiment, we also analyzed the localization of GM3 during chondrogenic differentiation from mesenchymal stem cells (MSCs). GM3 was transiently increased at days 21 and 28, and then declined at day 42 (Fig. 2). These results suggested that GM3 plays important roles in the late phase such as the hypertrophic process.

### Depletion of gangliosides in mice enhances articular cartilage repair

**Figure 3:**
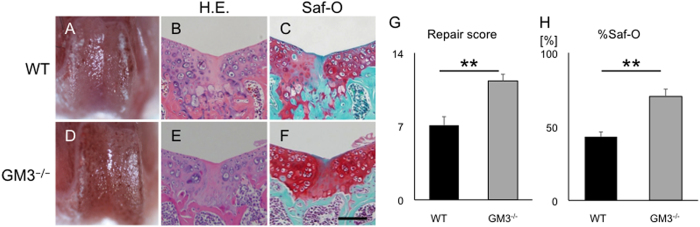
Eight weeks postoperative in 4-week-old mice. Macroscopic appearance of patella groove (**A**,**D**). Histology of the articular cartilage repair (**B**,**C**,**E** and **F**), the articular cartilage repair score (**G**), and %Saf-O (**H**). WT (**B**,**C**) and GM3^−/−^ mice (**E**,**F**) were stained with hematoxylin & eosin or Safranin-O staining. The scale bar shows 100 μm. All values are expressed as the mean ± SEM. (**P < 0.01).

We next analyzed articular cartilage repair at 8 weeks postoperative in 4-week-old mice (n = 9). GM3^−/−^ mice showed superior cartilage repair compared to WT mice (Fig. 3A–F). The repair score for the articular joint surface was significantly higher in GM3^−/−^ mice than WT mice (4-week-old: mean ± standard error of the mean (SEM); 7.1 ± 0.81 in WT mice versus 11 ± 0.63 in GM3^−/−^ mice [P = 0.00020], Fig. 3G). We also examined repair in 8-week-old mice. However, the repaired tissue in adult GM3^−/−^ mice was fibrous tissue at best. Therefore, we used 4-week-old mice in further experiments (8-week-old: mean ± SEM; 2.1 ± 0.24 in WT mice versus 5.1 ± 0.79 in GM3^−/−^ mice [P = 0.0079], Supplemental Fig. 2E). In addition, we conducted quantitative histomorphometry analysis in 4-week-old mice as previously reported^20^. The histomorphometry analysis clearly showed that the repaired tissue in GM3^−/−^ mice showed higher Safranin O staining than repaired tissue in WT mice. (%Saf-O: mean ± SEM; 43 ± 3.3 in WT mice versus 70 ± 4.9 in GM3^−/−^ mice [P < 0.0001]) (Fig. 3H). The high Safranin O staining of the hyaline cartilage matrix suggests that depletion of gangliosides may help improve the biomechanical properties.

### Immunostaining analysis shows that depletion of gangliosides suppresses the expression of hypertrophy-related genes

**Figure 4:**
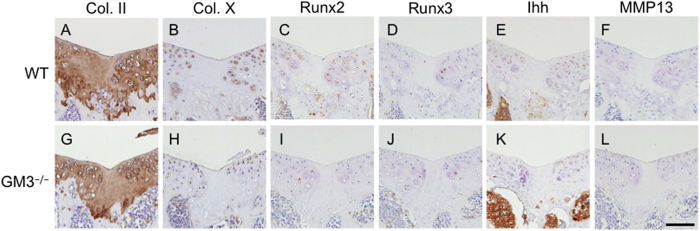
Immunostaining analysis of articular cartilage repair 8 weeks postoperative. Immunostaining of type II collagen (**A**,**G**) type X collagen (**B**,**H**), Runx2 (**C**,**I**), Runx3 (**D**,**J**), Ihh (**E**,**K**), and MMP13 (**F**,**L**). The scale bar shows 100 μm.

At 8 weeks postoperative, we found no significant difference in type II collagen staining between WT and GM3^−/−^ mice (Fig. 4A,G). Type X collagen in WT mice showed staining to some extent that surrounded the repaired tissue, whereas the staining of type X collagen in GM3^−/−^ mice was less intense (Fig. 4B,H). Although we could not detect the expression of Runt-related transcription factor (Runx)3 or Matrix metalloprotease (MMP)13 in repaired tissue, the expression of Runx2 and Indian hedgehog (Ihh) in GM3^−/−^ mice surrounding repaired tissue was suppressed compared to WT mice (Fig. 4C–F,I–L).

### Depletion of gangliosides does not affect the cell population

The above data revealed that articular cartilage injuries in GM3^−/−^ mice were repaired well due to suppression of chondrocyte hypertrophy surrounding the repaired tissue. This raised the possibility that depletion of gangliosides suppresses chondrocyte hypertrophy. To test this hypothesis, we performed a functional assay with MSCs and chondrocytes. The *in vitro* cell proliferation assay showed no significant difference between cells obtained from WT and GM3^−/−^ mice after 24 h (MSCs; mean ± SEM; 0.46 ± 0.027 in WT mice versus 0.51 ± 0.028 in GM3^−/−^ mice [P = 0.29], chondrocytes; 0.36 ± 0.013 in WT mice versus 0.37 ± 0.0049 in GM3^−/−^ mice [P = 0.28]; n = 6) (Supplemental Fig. 3). The wound healing assay also showed no significant difference between cells obtained from WT and GM3^−/−^ mice after 6 h (mean ± SEM; 0.12 ± 0.016 mm^2^ in WT mice versus 0.11 ± 0.0075 mm^2^ in GM3^−/−^ mice [P = 0.67], chondrocytes; 0.18 ± 0.013 in WT mice versus 0.16 ± 0.022 in GM3^−/−^ mice [P = 0.49]; n = 6) (Supplemental Fig. 4). Next, we analyzed how depletion of gangliosides affected the MSC population. Although the expression of type II collagen, Runx2, and Runx3 mRNA in MSCs was higher in GM3^−/−^ mice (Supplemental Fig. 5A–F), fluorescence activated cell sorting (FACS) analysis showed that the expression of stem cell markers was almost equal between WT and GM3^−/−^ mice (Supplemental Fig. 5G,H).

### Analysis of chondrogenic differentiation shows that depletion of gangliosides suppresses the expression of type X collagen via the Ihh pathway

**Figure 5:**
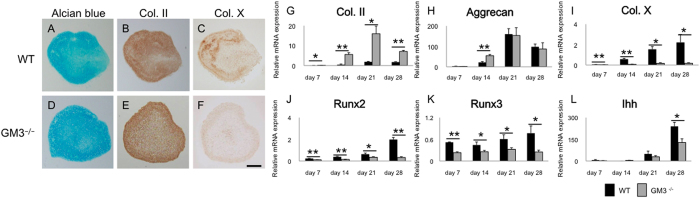
Chondrogenic differentiation from mouse MSCs. Alcian blue staining (**A**,**D**) and immunostaining for type II collagen (**B**,**E**) and type X collagen (**C**,**F**). The scale bars show 100 μm. Quantitative real-time reverse transcription-polymerase chain reaction analysis of the chondrogenic differentiation process (**G**–**L**) (**G**) type II collagen mRNA, (**H**) aggrecan mRNA, (**I**) type X collagen mRNA, (**J**) Runx2 mRNA, (**K**) Runx3 mRNA, (**L**) Ihh mRNA. All values are expressed as the mean ± SEM. (*P < 0.05, **P < 0.01).

Because the above data suggested that depletion of gangliosides did not affect the cell population, we investigated how depletion of gangliosides affected the chondrogenic program. In chondrogenic differentiation from MSCs, we observed no significant difference in Alcian blue staining or type II collagen (Fig. 5A,B,D,E). Staining for type X collagen was lower in GM3^−/−^ mice than in WT mice (Fig. 5C,F). During chondrogenic differentiation from MSCs, expression of chondrocyte-specific genes such as *type II collagen* and *aggrecan* mRNA increased with time in WT and GM3^−/−^ mice (Fig. 5G,H). At day 28, *type II collagen* mRNA was significantly upregulated in GM3^−/−^ mice (mean ± SEM; 1.7 ± 0.20 in WT mice versus 7.1 ± 0.54 in GM3^−/−^ mice [P < 0.0001] Fig. 4G), whereas *aggrecan* mRNA was not significantly different between WT mice and GM3^−/−^ mice (98 ± 13 in WT mice versus 87 ± 30 in GM3^−/−^ mice [P = 0.76] Fig. 5H). *Type X collagen* mRNA was significantly decreased in GM3^−/−^ mice (2.2 ± 0.78 versus 0.17 ± 0.047 [P = 0.041] Fig. 5I). The effect of depletion of gangliosides on expression of hypertrophy-related genes was also determined. *Runx2* and *3* and *Ihh* mRNA were significantly decreased in GM3^−/−^ mice (*Runx2*: 2.0 ± 0.19 in WT mice versus 0.33 ± 0.071 in GM3^−/−^ mice [P < 0.0001], *Runx3*: 0.77 ± 0.0.20 in WT mice versus 0.25 ± 0.050 in GM3^−/−^ mice [P = 0.047], *Ihh*: 240 ± 29 versus 130 ± 26 [P = 0.023], Fig. 5J–L).

### Analysis of hypertrophic differentiation in chondrocytes also shows that depletion of gangliosides suppresses the expression of type X collagen via the Ihh pathway

**Figure 6:**
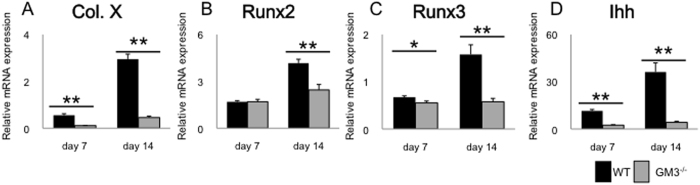
Quantitative real-time reverse transcription-polymerase chain reaction analysis of *in vitro* differentiation of murine primary chondrocytes. (**A**) type X collagen mRNA, (**B**) Runx2 mRNA, (**C**) Runx3 mRNA, (**D**) Ihh mRNA. All values are expressed as the mean ± SEM. (*P < 0.05, **P < 0.01).

To ask whether the role of gangliosides surrounding the repair site is cell-type specific for chondrocytes, we examined how the loss of gangliosides affected *in vitro* differentiation of murine primary chondrocytes to the terminal hypertrophic state. Interestingly, the expression of hypertrophy-related genes (*type X collagen, Runx family*, and *Ihh*) in GM3^−/−^ mice was significantly decreased after 14 days of induction (*type X collagen*: 2.9 ± 0.21 in WT mice versus 0.46 ± 0.064 in GM3^−/−^ mice [P < 0.0001], *Runx2*: 4.2 ± 0.25 in WT mice versus 2.5 ± 0.35 in GM3^−/−^ mice [P = 0.0025], *Runx3*: 1.6 ± 0.20 in WT mice versus 0.58 ± 0.072 in GM3^−/−^ mice [P = 0.0010], *Ihh*: 36 ± 5.8 versus 4.2 ± 0.73 [P = 0.00030], Fig. 6).

### Overexpression of GM3S enhances expression of hypertrophy-related genes in chondrocytes via the Ihh pathway

**Figure 7:**
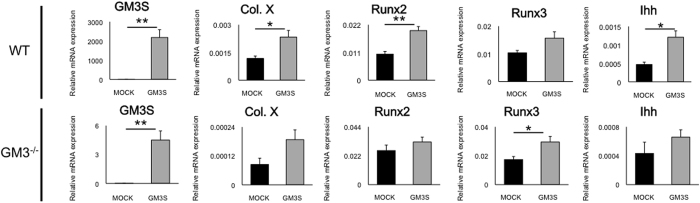
Association of the overexpression of GM3 synthase (GM3S) with hypertrophic-related genes. WT mice (**A–E**) and GM3^−/−^ mice (**F–J**). (**A,F**) GM3 synthase mRNA, (**B,G**) type X collagen mRNA, (**C,H**) Runx2 mRNA, (**D,I**) Runx3 mRNA, (**E,J**) Ihh mRNA. All values are expressed as the mean ± SEM. (*P < 0.05, **P < 0.01).

Finally, to confirm whether gangliosides regulate chondrocyte hypertrophy, we overexpressed GM3S in mouse chondrocytes. Transient transfection of GM3S in WT chondrocytes showed that *GM3S* mRNA expression was significantly upregulated in chondrocytes with the GM3S plasmid vector compared to those with the mock vector (mean ± SEM; mock: 0.0022 ± 0.0014, GM3S: 2200 ± 410 [P = 0.0046]; n = 4, Fig. 7A). *Type X collagen, Runx2*, and *Ihh* mRNAs were significantly increased in chondrocytes with the GM3S plasmid vector (*type X collagen*: mock: 0.0011 ± 0.00013, *GM3S*: 0.0023 ± 0.00036 [P = 0.049], *Runx2*: mock: 0.010 ± 0.0010, GM3S: 0.020 ± 0.0039 [P = 0.0071], *Ihh*: mock: 0.00047 ± 0.000064, *GM3S*: 0.0012 ± 0.00017 [P = 0.012], Fig. 7B,C,E). *Runx3* mRNA tended to increase in chondrocytes with the *GM3S* plasmid vector (Runx3: mock: 0.010 ± 0.00086 versus GM3S: 0.015 ± 0.0023 [P = 0.13], Fig. 7D). In addition, transient transfection of GM3S in GM3^−/−^ mouse chondrocytes showed no significant differences, but similar trends were observed (GM3S; mock: 0.0011 ± 0.0014, GM3S: 4.5 ± 0.95 [P = 0.0025] *type X collagen*: mock: 0.000084 ± 0.000026, GM3S: 0.00019 ± 0.000041 [P = 0.087], *Runx2*: mock: 0.026 ± 0.0042, GM3S: 0.032 ± 0.0036 [P = 0.32], *Runx3*: mock: 0.017 ± 0.0021 versus GM3S: 0.029 ± 0.0038 [P = 0.035], *Ihh*: mock: 0.00043 ± 0.00016, GM3S: 0.00066 ± 0.00010 [P = 0.30], Fig. 7F–J). Although, a direct comparison of these results with GM3 expression *in vivo* is extremely difficult, these results are consistent with the *in vivo* results showing that chondrocyte hypertrophy surrounding repaired tissue was regulated by the expression of gangliosides.

## Discussion

Time-sequential immunostaining after osteochondral injury revealed that GM3 expression was transiently enhanced surrounding the injury site during the late phase of the articular cartilage repair process. The distribution of GM3 expression strongly suggests that GM3 is involved in the cartilage healing process. Because our data showed that depletion of gangliosides suppresses chondrocyte hypertrophy, we were also interested in bone metabolism and/or remodeling. Thus, we investigated the depth of the cartilage in the repair site. However, the measurements were almost equal between WT and GM3^−/−^ mice (Supplemental Fig. 6). Based on these results, we believe that the effect of ganglioside depletion in bone is less than the effect in cartilage.

The expression pattern of only a few molecules has been investigated during the articular cartilage repair process. The chemokine stromal cell-derived factor 1 is transiently upregulated during the early phase and plays a role in recruitment of bone marrow stromal cells during articular cartilage repair^21^. Tenascin-C, a member of the extracellular matrix glycoprotein family, is also transiently increased during the early phase of articular cartilage repair and promotes chondrogenesis during the reparative process^22^. However, no previous reports have described transient enhancement during the late phase of the articular cartilage repair process. To the best of our knowledge, GM3 is the first molecule identified that was transiently enhanced during the late phase of the articular cartilage repair process.

Based on the transient enhancement of GM3 during the late phase after osteochondral injury, we speculated that gangliosides play important roles in late chondrogenic differentiation including hypertrophy rather than cell migration and proliferation. To test our hypothesis, we first evaluated cell migration and proliferation of cells from WT and GM3^−/−^ mice. Our results predictably revealed no difference in cell migration or proliferation, whereas the depletion of gangliosides suppressed chondrocyte hypertrophic differentiation. Chondrocyte hypertrophy, which eventually results in ectopic ossification, is an unresolved issue in cartilage tissue engineering^23^,^24^. Chondrogenesis without chondrocyte hypertrophy is required to organize and maintain reparative hyaline cartilage. Manipulation of GM3 function may overcome this issue and avoid chondrocyte hypertrophy with strict regulation.

Although the depletion of gangliosides accelerates cartilage degradation^15^, GM3^−/−^ mice unexpectedly showed accelerated articular cartilage repair in our current study. As mentioned above, inhibition of chondrocyte hypertrophy enhances cartilage regeneration. Therefore, this inhibition may become a therapeutic strategy for articular cartilage repair^5^,^25^. The roles of hypertrophic differentiation during articular cartilage repair differ from those during cartilage degradation. Previous reports using genetically modified mice showed that enhancement of chondrocyte hypertrophy was frequently associated with a higher incidence of OA or acceleration of OA^26^,^27^,^28^. On the other hand, hypertrophic differentiation is a common compensatory reaction against mechanical stress to prevent cartilage degeneration^29^. Regarding the OA pathogenesis in GM3^−/−^ mice, we speculate that inhibition of chondrocyte hypertrophy disrupted resistance to mechanical stress rather than accelerated cartilage repair^15^,^30^.

Ihh, a member of the Hedgehog family of proteins, is essential for growth plate formation and chondrocyte maturation via Runx2 and Runx3 expression^31^. Regarding OA pathogenesis, a genetic study in mice showed that conditional deletion of Ihh in chondrocytes attenuates OA progression, suggesting that blockade of Ihh signaling may be useful as a therapeutic approach to prevent or delay cartilage degeneration^32^. However, Ihh gene deletion is currently not a therapeutic option as it is lethal in animals^33^. In the current study, GM3^−/−^ mice showed enhanced articular cartilage repair via suppression of Ihh signals. GM3^−/−^ mice develop normally without serious systemic effects^15^,^34^, suggesting that the ganglioside-mediated approach to modifying the Ihh pathway is a novel gene therapy strategy that may have less influence on systemic conditions.

Several limitations to this study must be considered when interpreting the present results. First, although we used the osteochondral injury model for the analysis of the articular cartilage repair process, how the MSCs from the subchondral bone are involved in the current study remains unclear. Because identification of the cells in the articular cartilage repair sites is difficult, the cells that mainly produce the matrix were assumed to be chondrocytes surrounding repaired tissue. To ask whether MSCs participate in this phenomenon, we examined chondrogenic differentiation from murine MSCs, and results showed that depletion of gangliosides suppressed chondrocyte hypertrophy in MSCs. In addition, depletion of gangliosides in chondrocytes suppressed hypertrophic differentiation. We were unable to identify the origin of chondrocytes in the current study. However, taking into account the transient enhancement of GM3 surrounding repaired tissue in the late phase, we speculate that the GM3-positive cells mainly differentiated into hypertrophic chondrocytes. This point should be addressed in future studies. Second, we investigated the articular cartilage repair process using young mice because we and others have previously reported that the healing potential of adult C57BL/6 mice is limited^10^,^35^,^36^. Although the mechanisms of articular cartilage repair may vary with age, we believe that the healing mechanisms of articular cartilage in immature mice will provide important insights that will be applicable to the manipulation of cartilage healing in adults. Third, how gangliosides regulate the expression of chondrogenic-related genes remains unclear. Gangliosides are widely distributed in vertebrate plasma membranes and act as modulators of various types of transmembrane signaling. Because gangliosides do not have any specific ligands, conducting functional analysis using ganglioside-null mice is extremely difficult. The chondrocytes surrounding repaired tissue show increased tendency toward to hypertrophic differentiation (Fig. 4), suggesting that gangliosides specifically act when the cellular metabolic activity is enhanced. However, from the current study, we can conclude that gangliosides participate in the articular cartilage repair process by suppressing chondrocyte hypertrophy. This issue should be addressed in future studies.

To the best of our knowledge, this is the first study to reveal that gangliosides regulate hypertrophic differentiation of chondrocytes. Depletion of gangliosides enhanced the articular cartilage repair process via the Ihh pathway. Although a future study is needed to ensure consistency with OA, inhibition of hypertrophic differentiation may be a future strategy for accelerating cartilage repair.

## Methods

### Animals and generation of gene-deleted mice

GM3^−/−^ mice were generated as described previously^34^. C57BL/6 mice were purchased from Japan SLC, Inc. (Shizuoka, Japan) and used as WT mice. Mice were utilized after acclimatization for 7 days following transportation^37^. All purchased mice returned to their normal behavior within 24 h after transportation. The mice used in the current study were housed in a temperature- and humidity-controlled environment under 12-h light/12-h dark conditions and fed a standard rodent diet. All animal experiments were performed in accordance with protocols approved by the Institute of Animal Care and Use Committee of the Hokkaido University Graduate School of Medicine.

### Generation of articular cartilage injuries

Articular cartilage full-thickness injuries were generated in 4-week-old and 8-week-old female mice^10^. Briefly, the two ends of 21-G needles were cut to create an outer cylinder that was adjusted to be approximately 300 μm shorter than a 27-G needle, and this original device was used to create the injury. Longitudinal full-thickness injuries along the femoral axis were made in the patellar groove using the ends of the needles of the device described above.

### Tissue processing and histology

At each postoperative period, the knee joints were fixed with 10% formalin (WAKO, Tokyo, Japan), and then decalcified with ethylenediaminetetraacetic acid, embedded in paraffin, and sectioned at 5-μm thicknesses. Histological sections were made as previously reported^10^,^35^. Briefly, axial sections were made at three points per knee joint to make sure the articular cartilage repair site was included. The first section was 100 μm proximal to the intercondylar notch, the second one was another 100 μm proximal to the first section, and the third one was 100 μm proximal to the second section. The sections at each point were used for histomorphometry and scoring. All slides were analyzed using an Olympus DP72 camera and DP2-BSW software (Olympus, Tokyo, Japan).

### Macroscopic and histological evaluations of articular cartilage repair

The animals were euthanized for further investigation at 8 weeks postoperative. The knee samples were photographed using a Nikon SMZ745T (Nikon, Tokyo, Japan). Each section was stained with hematoxylin & eosin and Safranin-O staining. The histological score for joint surface repair of each section was evaluated as previously reported^10^,^38^. The scoring was performed independently by two blinded observers. Quantitative histomorphometry of Safranin O-stained sections was performed using Image J software (National Institutes of Health, Bethesda, MD, USA) as previously reported^20^. The total region of the articular cartilage repair site was defined and measured as the total repair tissue volume. The percent Safranin O-stained repaired tissue in the total soft tissue volume (%Saf-O) was obtained. The depth of the cartilage in the repaired tissue was measured at 8 weeks postoperative using Image J software. All data are presented as the means ± standard error of the mean (SEM).

### Isolation of MSCs and chondrocytes

MSCs were harvested from the compact bone of 2-week-old mice as previously reported^39^ and cultured in alpha Modified Eagle Minimum Essential Medium (Sigma, St. Louis, MO) with 2 mM l-glutamine, 1% antibiotics (WAKO), and 10% fetal bovine serum (NICHIREI, Tokyo, Japan). Immature mouse chondrocytes were obtained from the knee joints of newborn mice as previously described^40^. Chondrocytes were cultured in Dulbecco’s modified Eagle’s medium (WAKO) with 10% fetal bovine serum (NICHIREI) and 1% antibiotics (WAKO).

### Quantification of GSL-glycans in the chondrocytes by mass spectrometry

GSLs were recovered from mouse chondrocyte pellets by chloroform-methanol extraction, and GSL-glycans were released by endoglycoceramidase digestion as previously reported^15^. Released intact GSL-glycans were subjected to a glycoblotting procedure and matrix-assisted laser desorption ionization-time-of-flight/time-of-flight mass spectrometry analysis.

### *In vitro* cell viability assay

To investigate the effects of ganglioside deficiency on cellular viability, mouse MSCs or chondrocytes were cultured in 96-well tissue plates in growth medium. The number of viable cells at each time was counted with Cell Counting Kit-8 (Dojindo Laboratories, Kumamoto, Japan) by quantification of the absorbance at 450 nm.

### *In vitro* wound healing assay

To investigate cell motility, an assay described previously was used with slight modifications^41^. Briefly, mouse MSCs or chondrocytes were cultured to confluent monolayers in 6-well tissue plates in growth medium and subsequently wounded by uniform scratching with a pipette tip. This initial wounding and the movement of the cells into the blank field of the scratch line after 6 h were photographically documented using an Olympus IX71 microscope (Olympus). The migration rate was calculated as the reduction area, which remained cell-free after scratching between both borderlines. Images were obtained using Image J software.

### QPCR

Total RNA was extracted from the samples using the RNeasy Mini kit (Qiagen, Hilden, Germany). For complementary DNA synthesis, 1.0 μg RNA was reverse transcribed using random hexamer primers (Promega, Tokyo, Japan) and ImProm II reverse transcriptase (Promega). QPCR was performed using a Thermal Cycler Dice Real Time System II (Takara Bio Inc., Otsu, Japan). Signals were detected using SYBR Premix Ex Taq II (Takara) with gene-specific primers (Supplementary Table 1). The relative mRNA expression of each targeted gene was expressed as the Ct value of each gene normalized to the Ct value of GAPDH by using the δδCt method^42^.

### Flow cytometry

We quantitatively investigated the population difference between WT and GM3^−/−^ mice. MSCs (1 × 10^6^) were stained with FITC-conjugated anti-mouse CD45 (eBioscience, USA), PE-conjugated anti-mouse CD29 (eBioscience), or PE-conjugated CD86 antibodies (eBioscience). Cell surface marker expression was analyzed by flow cytometry (FACSCanto, BD Biosciences, San Jose, CA, USA).

### Chondrogenic differentiation of MSCs

MSCs (1 × 10^6^) were placed in polypropylene tubes and centrifuged at a speed of 300 × *g* for 6 min. The pellets were cultured in 1 ml complete chondrogenic medium consisting of Dulbecco’s modified Eagle’s medium (Life Technologies, Gaithersburg, MD, USA) supplemented with 10^−7^ M dexamethasone (Sigma), 1% insulin-transferrin-sodium selenium (Sigma), 50 μM ascorbate-2-phosphate (WAKO), 50 μg/ml proline (WAKO), 20 ng/ml transforming growth factor-β3 (Life Technologies), and 1% antibiotics (WAKO)^39^. The polypropylene tubes were maintained at 37 °C in a 5% CO_2_ incubator, and the medium was changed every 3 days. The pellets were used for immunostaining or qPCR at each time. Data were normalized to the average mRNA level at day 0 (set at 1) and are presented as means ± SEM.

### Hypertrophic differentiation of chondrocytes

Mouse chondrocytes were seeded in 24-well tissue culture plates and cultured in Dulbecco’s modified Eagle medium–F-12 (WAKO) supplemented with 10% fetal bovine serum (NICHIREI), 1% ITS Media supplement (containing insulin, transferrin, and selenous acid; Sigma), and 50 μg/ml ascorbate-2-phosphate (WAKO) for the indicated times. The cells were maintained at 37 °C in a 5% CO_2_ incubator with medium changes every 48 h. Data were normalized to the average mRNA level at day 0 (set at 1) and are presented as means ± SEM.

### Immunohistochemistry

Sections were deparaffinized, and endogenous peroxidase activity was quenched. Sections were incubated with rabbit polyclonal antibodies against rat type II collagen (1:50; Daiichi Fine Chemical, Toyama, Japan), type X collagen (1:200; LSL, Tokyo, Japan), Runx2 (1:100; Santa Cruz Biotechnology, Waltham, MA, USA), Runx3 (1:100; Cell Signaling Technology, Beverly, MA, USA), Ihh (1:250; Abcam, Cambridge, MA, USA), and MMP-13 (1:100; Abcam). After washing with phosphate-buffered saline three times, the samples were incubated with a biotinylated secondary antibody and visualized by the precipitation of diaminobenzidine (DAKO, Copenhagen, Denmark).

### Preparation of frozen sections and immunostaining for GM3

We prepared frozen sections at each time point from the samples^43^. Sections were incubated with the primary antibody anti-GM3 (M2590, 1:100; Cosmo Bio, Tokyo, Japan), and staining was visualized with an Alexa 594-conjugated anti-rabbit IgM antibody (1:1000; Life Technologies)^17^. Negative control sections were incubated with iso-type matched control serum (1:100; DAKO). Nuclei were stained with DAPI (Dojindo, Kumamoto, Japan). All slides were analyzed with confocal laser scanning microscopy (Olympus Fluoview FV300).

### Alcian blue staining

The cells were rinsed with phosphate-buffered saline twice, fixed with 95% methanol, and then stained with Alcian blue (pH 2.5, Muto Pure Chemicals, Tokyo, Japan) for 60 min. Then, cells were washed with 3% acetic acid for 3 min and photographed.

### Plasmid preparation and transient transfection

Plasmid vectors were designed by and purchased from Ori-Gene (Rockville, MD). The expression vector for GM3S was St3gal5 (NM_001035228). The mouse cDNA clone was OriGene MC209401, and the mock control vector was pCMV6-Entry (OriGene PS100001). Mouse chondrocytes were seeded in 24-well tissue culture plates. After the chondrocytes reached subconfluency, the cells were transfected using Lipofectamine LTX (Invitrogen, Carlsbad, CA, USA) according to the manufacturer’s instructions. After 48 h, the cells were used for analysis.

### Statistical analysis

Mean histological scores were statistically compared with non-parametric Wilcoxon tests between pairs of groups. Statistical comparison of data between the two groups of samples was performed with unpaired t tests. Significance was accepted with a P value < 0.05. All statistical analyses were done using statistical software JMP Pro 11.0 (SAS Institute, Cary, NC, USA).

## Additional Information

**How to cite this article:** Matsuoka, M. *et al*. Depletion of Gangliosides Enhances Articular Cartilage Repair in Mice. *Sci. Rep.* **7**, 43729; doi: 10.1038/srep43729 (2017).

**Publisher's note:** Springer Nature remains neutral with regard to jurisdictional claims in published maps and institutional affiliations.

## Supplementary Material

Supplemental Figure
