# Supplementary material for: Depletion of Gangliosides Enhances Articular Cartilage Repair in Mice
Source: Sci Rep. 2017 Mar 2;7:43729. doi: 10.1038/srep43729 (PMC5333092; doi:10.1038/srep43729)
Supplement: Supplemental Figure [file srep43729-s1.pdf]

Depletion of Gangliosides Enhances Articular Cartilage Repair in Mice

Masatake Matsuoka[1], MD, PhD; Tomohiro Onodera[1,*], MD, PhD; Kentaro Homan[1], MS, RPT; Fumio Sasazawa[1], MD, PhD; Jun-ichi Furukawa[1], PhD; Daisuke Momma[1], MD, PhD; Rikiya Baba[1], MD; Kazutoshi Hontani[1], MD; Zenta Joutoku[1], MD; Shinji Matsubara[1], MD; Tadashi Yamashita[2], DVM, PhD; Norimasa Iwasaki[1], MD, PhD

[1]Department of Orthopaedic Surgery, Hokkaido University Graduate School of Medicine, Sapporo, Japan

[2]Laboratory of Biochemistry, Azabu University, Graduate School of Veterinary Medicine, Sagamihara, Japan

Supplemental figure legends

Supplemental Figure 1. Quantitative analysis of GSL-glycans in cultured mouse chondrocytes.

Supplemental Figure 2. Eight weeks postoperative in 8-week-old mice. Histology of articular cartilage repair (A-D) and the articular cartilage repair score (E). Sections from WT (A-B) and GM3$^{-/-}$ mice (C-D) were stained with hematoxylin & eosin or Safranin-O. The scale bar shows 100 µm. All values are expressed as the mean ± SEM. (**P < 0.01).

Supplemental Figure 3. Proliferation assay. A) MSCs, B) chondrocytes. The number of viable cells quantified by measuring the absorbance at 450 nm. All values are expressed as the mean ± SEM.

Supplemental Figure 4. Wound healing assay. A) MSCs, B) chondrocyte. The migration rate was calculated as the reduced area that remained cell free between both borderlines following scratching. All values are expressed as the mean ± SEM.

Supplemental Figure 5. Quantitative real-time reverse transcription-polymerase chain reaction analysis of MSCs. (A) type II collagen mRNA, (B) aggrecan mRNA, (C) type X collagen mRNA, (D) Runx2 mRNA, (E) Runx3 mRNA, (F) Ihh mRNA. All values are expressed as the mean ± SEM. (**P < 0.01). Flow cytometry analysis of MSCs (G-L). The expression of each antigen (red) is shown together with the corresponding isotype control (blue). Numbers indicate the percentage of cells in the indicated regions. FACS results show that these cells are homogenously positive for the mesenchymal marker CD29 (G, J) but negative for the hematopoietic marker CD45 (H, K) and co-stimulating molecule CD86 (I, L).

Supplemental Figure 6. Measurement of the articular cartilage repair site in 4-week-old mice at 8 weeks postoperative. All values are expressed as the mean ± SEM.

Supplemental Table 1. Gene-specific primers used for real-time PCR analysis.

Supplemental Figure 1

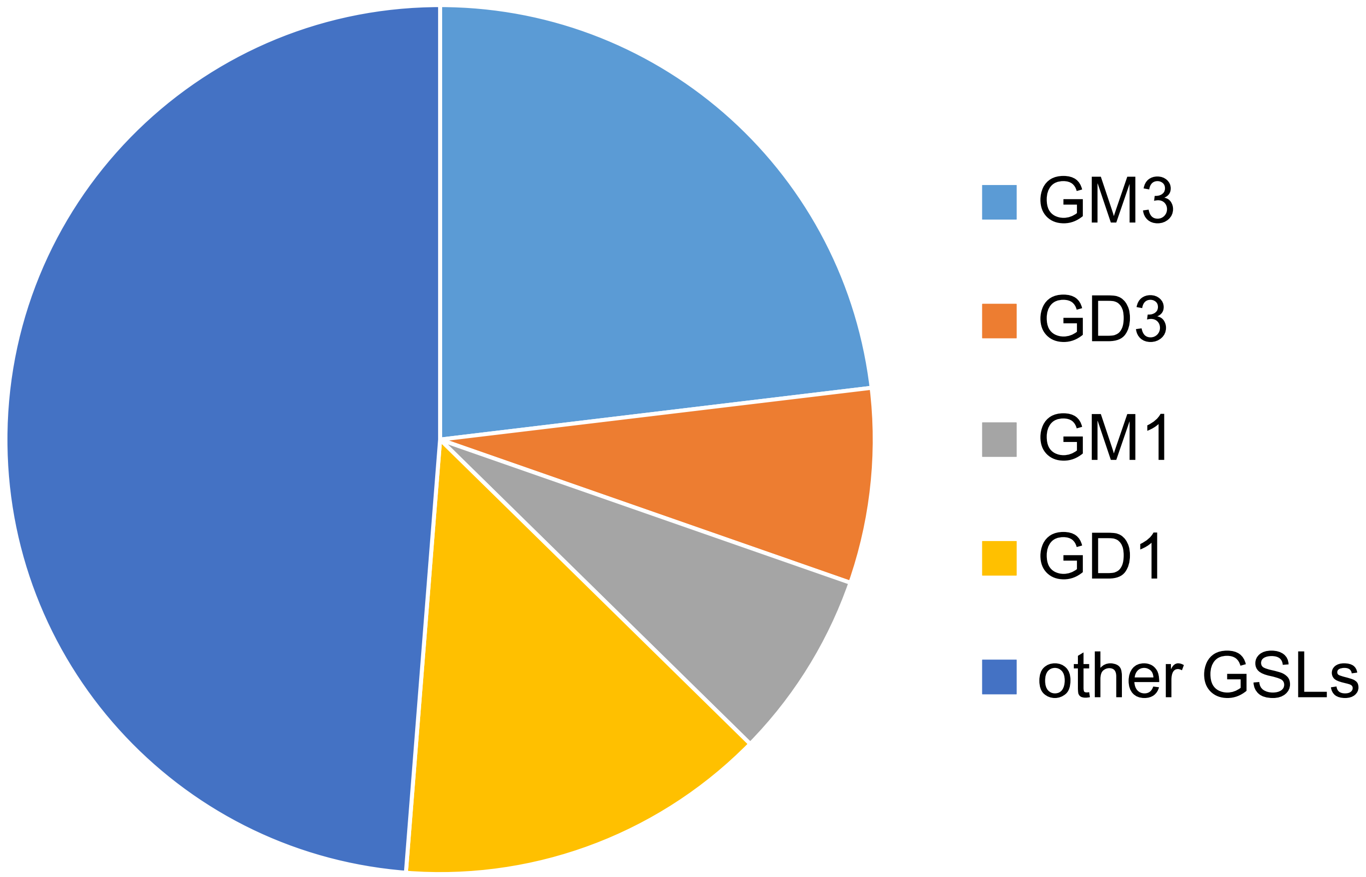

Supplemental Figure 2

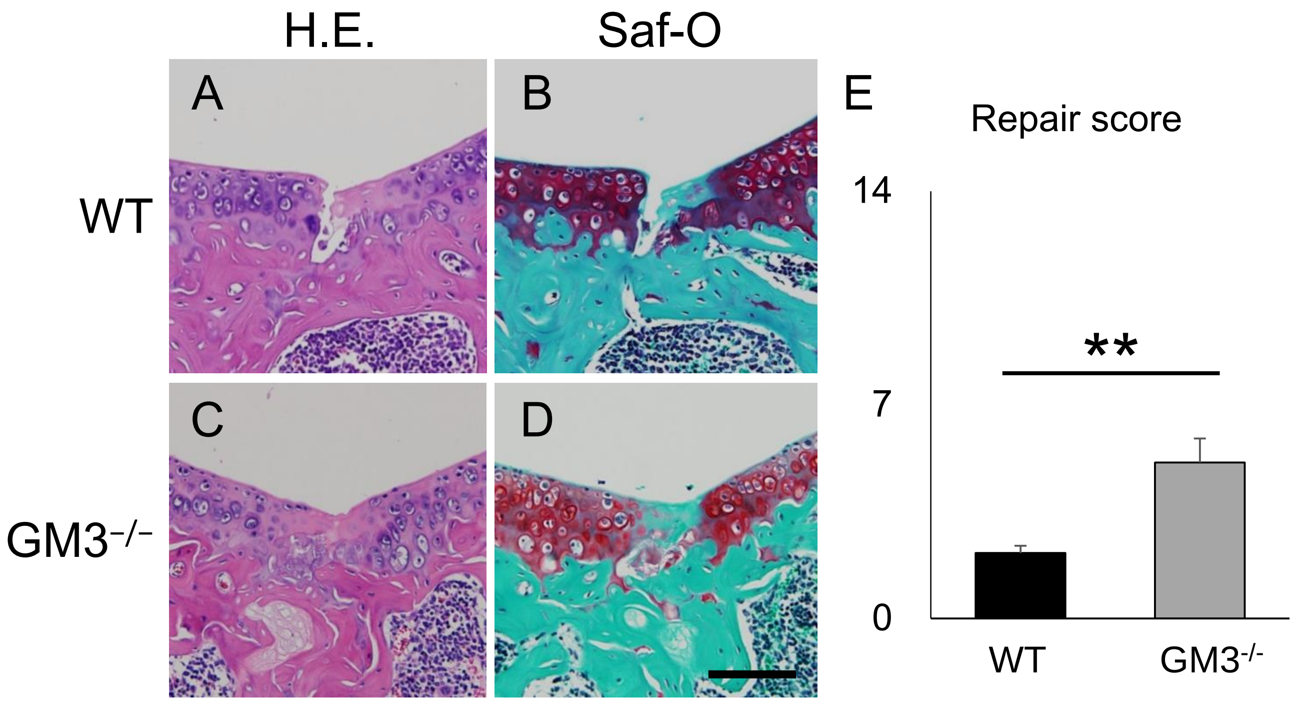

Supplemental Figure 3

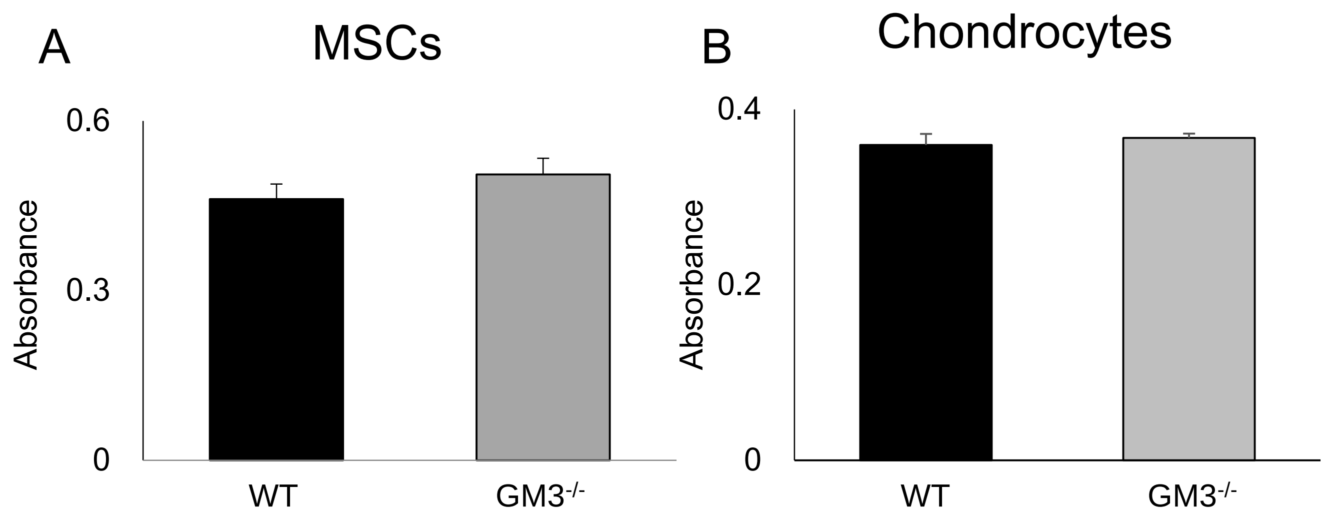

Supplemental Figure 4

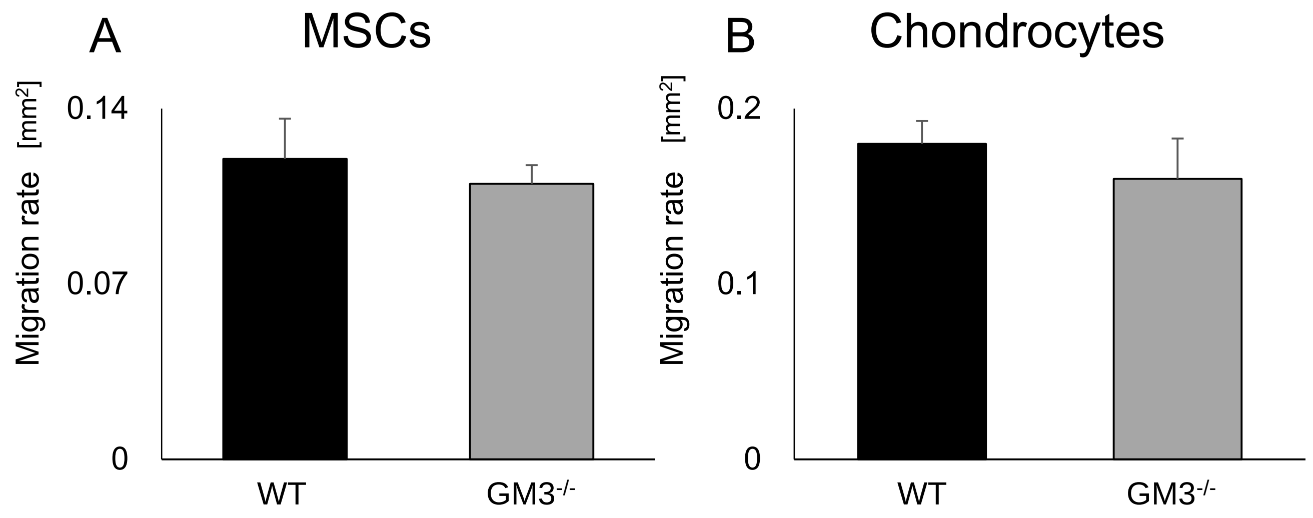

Supplemental Figure 5

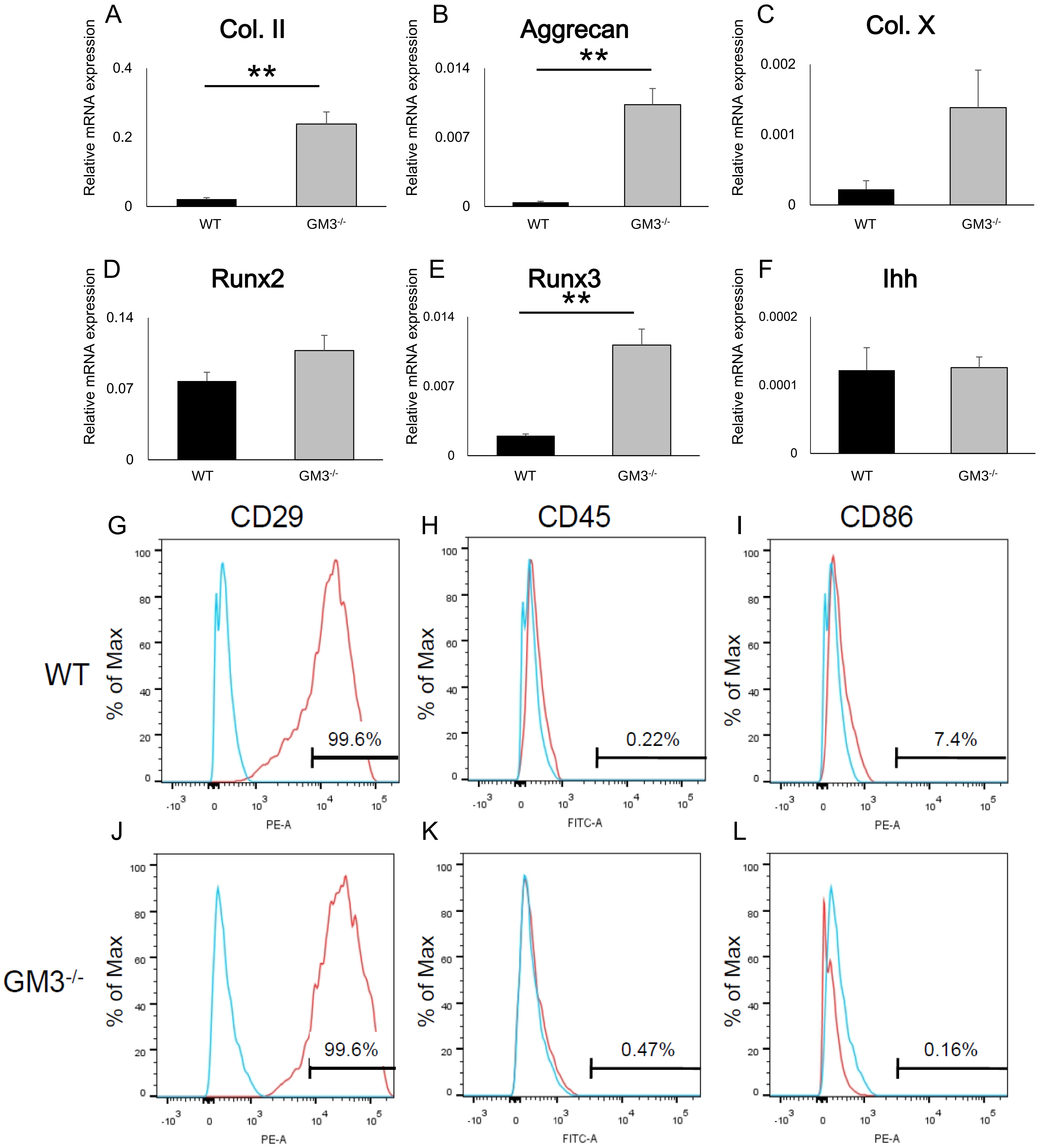

Supplemental Figure 6

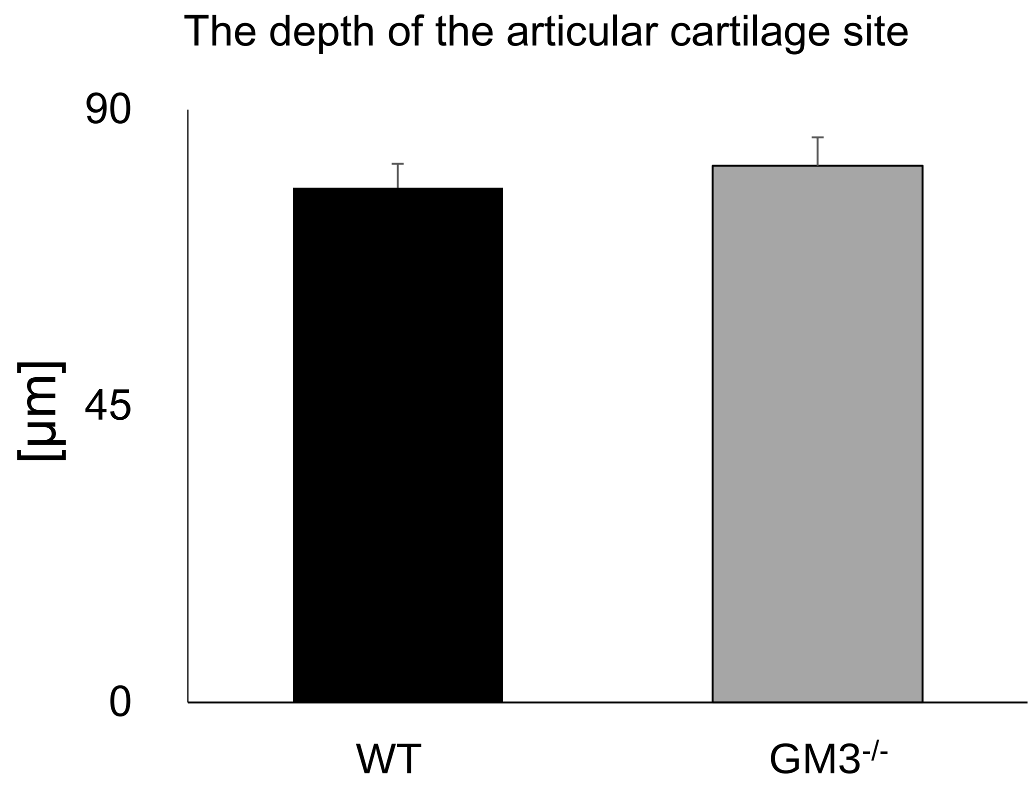

Supplemental Table 1

Gene	Sequence	
Type II collagen	Forward	AGGATGGCTGCACCAAACAC
	Reverse	TGTCCATGGGTGCGATGTC
Type X collagen	Forward	AGCCCCAAGACACAATACTTCATC
	Reverse	TTTCCCCTTTCCGCCCATTCACAC
Aggrecan	Forward	CCCTCACCCCAAGAATCAAG
	Reverse	GGATAGTTGGGGAGCGACAC
Runx2	Forward	GATGGGACTGTGGTTACCG
	Reverse	GGTGAAACTCTTGCCTCGTC
Runx3	Forward	CAGGTTCAACGACCTTCGATT
	Reverse	GTGGTAGGTAGCCACTTGGG
Inh	Forward	CTCTTGCCTACAAGCAGTTCA
	Reverse	CCGTGTTCTCCTCGTCCTT
GM3 synthase	Forward	ATGCCAAGTGAGTTCACCTCT
	Reverse	ACTCCAAATGCAACCAACGTG
GAPDH	Forward	ACTTTGTCAAGCTCATTTCC
	Reverse	TGCAGCGAACTTTATTGATG
